# Content Disputes in Wikipedia Reflect Geopolitical Instability

**DOI:** 10.1371/journal.pone.0020902

**Published:** 2011-06-22

**Authors:** Gordana Apic, Matthew J. Betts, Robert B. Russell

**Affiliations:** 1 Cell Networks, University of Heidelberg, Heidelberg, Germany; 2 Cambridge Cell Networks Ltd., St. John's Innovation Centre, Cambridge, United Kingdom; University of Maribor, Slovenia

## Abstract

Indicators that rank countries according socioeconomic measurements are important tools for regional development and political reform. Those currently in widespread use are sometimes criticized for a lack of reproducibility or the inability to compare values over time, necessitating simple, fast and systematic measures. Here, we applied the ‘guilt by association’ principle often used in biological networks to the information network within the online encyclopedia Wikipedia to create an indicator quantifying the degree to which pages linked to a country are disputed by contributors. The indicator correlates with metrics of governance, political or economic stability about as well as they correlate with each other, and though faster and simpler, it is remarkably stable over time despite constant changes in the underlying disputes. For some countries, changes over a four year period appear to correlate with world events related to conflicts or economic problems.

## Introduction

Recent studies have demonstrated the power of the World Wide Web to provide fascinating insights into a wide range of subjects. For example, Google search terms are an excellent predictor of influenza outbreaks [1], it is possible to predict book partisan loyalties in the United States by an analysis of Amazon recommendations [2], and new Web 2.0 utilities such as Twitter can play significant roles in world political events [3]. As much of the information on the Web is cross-linked, tools from multiple disciplines for the study of networks can be used.

Possibilities for exploiting networks in the biological [4], physical [5] & social sciences [6] as well as in the commercial world (e.g. [7]) have produced a vibrant discipline which exploits networks analytically and predictively. Many networks have been found to be scale free which has implications for error and attack tolerance [8], and existing connections within a network can be used predictively; for instance, social networks have been used to predict consumer purchasing preferences [9]. More abstract predictions are also possible, for example, knowledge of collaborations and time-commitment within networks of researchers can predict the fate of research communities [10].

Existing connections in biological networks have been used to suggest new molecular interactions (e.g. [11]), and other phenomena such as the correlation between protein network centrality and gene deletion lethality [12]. However, probably the most exploited concept in these networks is that of “guilt by association” [13], [14]. Here, molecules that are poorly understood can be assigned functions similar to better studied molecules following high-throughput or genome-scale interaction experiments that show them to be linked together. For example, if a new molecule is found by experiments to be associated with molecules involved in (say) DNA repair, then one can predict with some confidence a DNA repair role for the new molecule. The confidence of the prediction goes up when there are multiple associations (links to ten molecules involved in DNA repair is better than a single link). It is this concept that we exploit here, but using instead the network of information contained within Wikipedia to create a geopolitical indicator based on disputes among its contributors.

Wikipedia is an online encyclopedia consists of millions of pages of information on every conceivable subject. These pages are extensively cross-linked to each other, providing a vast information network. The content is owned or controlled by no one, and that the many millions of pages contained can be edited by anybody. Despite what might be considered a chaotic approach, the accuracy of Wikipedia has been argued to be close to that of Encyclopedias constructed by experts [15]. Naturally conflicts arise when material is sensitive, and the site provides a means of open discussion for eventual resolution. To inform readers that the pages do not yet correspond to the established standards on neutrality (NPOV or a neutral point of view), they are labeled as ‘NPOV disputes’ (e.g. *The neutrality of this article is disputed*), and linked to a page explaining how disputes should be resolved.

Here, we investigated the ranking of countries according to the number of disputed pages that linked to the main page for the country itself. This is logical as much of the content of Wikipedia is dedicated to geographical, historical and political information which in turn is linked to pages for individual countries, which are seldom disputed themselves. We describe the Wikipedia Dispute Index which scores and ranks countries according to neutrality disputes, and show that it agrees with two other indicators of political stability about as well as they agree with each other. We also show that changes in the indicator over a four year period correlate with some global events that would be expected to impact on regional stability.

## Results and Discussion

**Figure 1:**
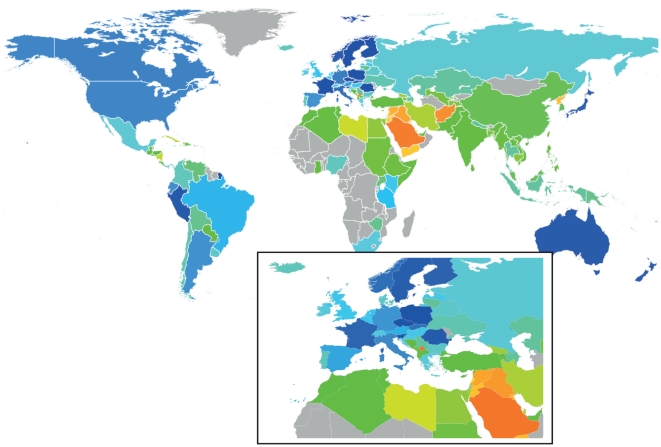
Mercator projection of the world colored according the Wikipedia dispute index. Colors traverse the spectrum from red (many more disputes than average) to blue (many fewer than average). Countries having too few disputes to be considered are colored grey. Note that French Territories (e.g. French Guyana, Mayotte, etc.) are colored according to France.

The indicator (the Wikipedia Dispute Index) considers the frequency of disputed pages linked to a country compared to that expected on average (see Methods). The world heat-map constructed using this measure (Figure 1) suggests that disputes in Wikipedia do correlate with regional instabilities across the world. Of the 138 (of 497) countries/regions with sufficient data to compute the indicator with confidence, the most disputed are parts of the middle east followed by other regions such as Kosovo, Bosnia & Herzegovina and North Korea (Figure 1; Table S1). At the other extreme, countries in North America and Western Europe are the least disputed, with most other countries occupying a middle range.

There are certain exceptions, such as Poland, Peru or Romania that have fewer disputes than might be expected. Inspection suggests that these outliers are likely to do with fewer pages in English than languages of the region; the Polish Wikipedia is the fourth largest, the Spanish, seventh. The picture for Peru (and the rest of South America) changes when one considers the Spanish version of Wikipedia (Figure S1), though only the English Wikipedia covers the globe to a useful degree (138 countries compared to 24 for German, 30 for French, 50 for Spanish). There are also many countries (see grey in Figure 1 and Figure S1) where there are currently too few pages or disputes to compute our measure with confidence. A consideration of other languages could lead to a more comprehensive list, though lack of internet access locally and/or diaspora in better connected countries could be an additional limitation (e.g. see Africa in Figure 1 and Figure S1).

The biggest contributors to the indicator tend to be disputes over current or historical events or individuals that vary according to different political views. However, other contributing factors are less intuitive, for instance, the disputed page “Adultery” is linked to several Middle-eastern and South American countries. There are also what appear to be spurious links, or those that can only loosely be linked to the countries of interest. For example, the page related to the football club “FC Aarau” was disputed in late 2010, and linked to Moldova owing to a Moldovese player. However, such links appear to be exceptions forming a background of disputes that likely contributes equally to all countries (see Methods).

**Figure 2:**
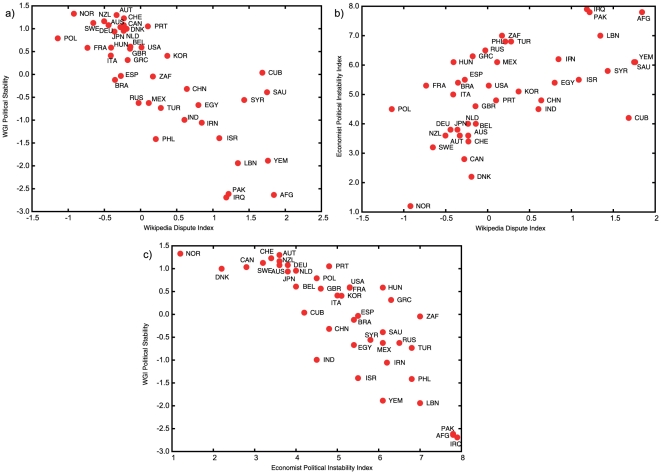
Plots comparing the Wikipedia Dispute Index (X axis) to a) the World Bank Policy Research Aggregate Governance Indicator (WGI) for political stability [**13**] (R = −0.781), and b) the Economist Intelligence Unit 2009 political instability index [**14**] (R = 0.641). The third plot c) shows the two other indicators plotted against each other (R = −0.732). Only countries with more than 100 disputes are shown for clarity.

There are many other governance, economic or political indicators in common use (e.g. [16], [17]). These are subject to criticisms such as the inability to compare changes over time, biases towards particular experts' opinions, or disparate and/or subjective data sources [18]. Our dispute index agrees with other indicators of political stability/instability [16], [17] about as well as they agree with each other (Figure 2; Figure S3) and the correlation improves with increasing data stringency (Figure S2), suggesting that index should improve as Wikipedia grows in size. Considering the components of known indicators (see Methods), the best agreement to our indicator are to the “Underlying Vulnerability” metric devised by the Economist Intelligence Unit [13], and to “Voice and Accountability” from the World Bank Governance Indicators [16] (Figure S3), which are perhaps the metrics most similar to the tension captured within Wikipedia disputes. The other indicators vary considerably in what they measure, and how they are calculated, but typically they are based on combining various political or economic metrics, questionnaires and opinions. The dispute index is not free from subjectivity as it is derived from a web site with thousands of contributors with differing opinions. However, it is easy to calculate, and does not rely on complex data gathering or the solicitation of experts. It also changes over time seemingly in concert with major world events (see below).

**Figure 3:**
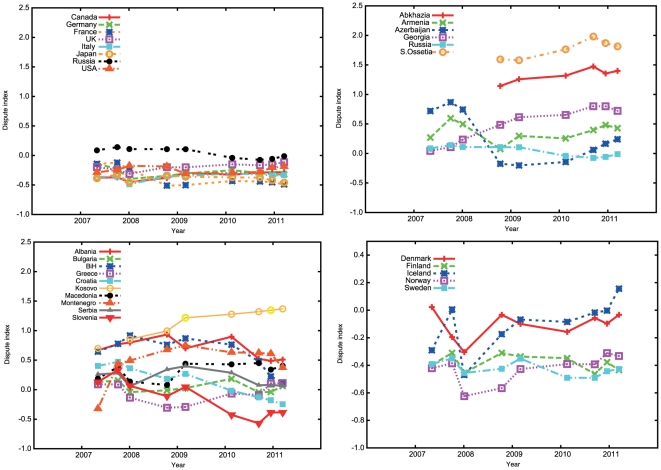
Values of the dispute index over a 3 year period for a) the G8 countries, b) countries in the Caucasus, c) the Balkans and d) Nordic countries. Only those countries and points are shown where the number of disputes is above our background threshold of 20 (see Methods). Note that the scale on the y-axis is different in d) compared to the rest.

A natural question is how long this indicator will be useful in the wake of the constant editing and conflict resolution efforts of contributors. There are pages that are difficult to resolve despite months or even years of discussion, but many are resolved. For instance, the page named “Islam and Antisemitism” lost its disputed status in 2010, whereas the page “Demographics of Kosovo” created in February 2007 picked up a dispute in mid-2008 and remains disputed at the time of writing. However, despite many changes in the pages in dispute, the rankings are relatively stable over time, for instance when considering the G8 countries (Figure 3). This is remarkable considering the drastic changes in the underlying disputed pages: on average, only 7.8% of disputed pages linking to countries were common when comparing datasets for August 2010 and April 2007.

There are nevertheless revealing changes over the time period we studied (Figure 3). For instance for the Balkan or Caucasus regions, changes appear roughly in line with political events: values for South Ossetia, Abkhazia and Georgia increased during and after the 2008 war; Kosovo increased after the 2008 declaration of independence. Trends go both ways: for instance Slovenia shows a steady decrease correlating perhaps with EU integration (its value goes towards those for Western EU members). The indicator for Iceland increased slightly relative to other Nordic countries during the recent Economic crisis (a slight upward trend is also seen recently for Greece in the Balkans plot). However, such changes are not always apparent: values for Middle Eastern and North African countries, for example, were stable over the recent revolutionary period. To provide the means to chart changes over time, we have created a web resource with a version of the map in Figure 1 and cross references that will be updated weekly (see www.disputeindex.org).

It is remarkable that so simple a metric can agree so well with more complex measures of political and economic stability. We do not mean to suggest that this indicator could replace existing metrics since the issues mentioned above related to sparse data and language currently preclude this possibility. However, this work does demonstrate that information contained within resources like Wikipedia can be used in interesting and useful new ways that can ultimately complement more arduous metrics. Further systematic analyses of vast information networks now available on the Web with the tools and expertise of multiple disciplines will clearly continue to impact on many subjects.

## Methods

### Search strategy

Pages below and in the text refer to the English version of Wikipedia (URLs beginning en.wikipedia.org/wiki/). We obtained a country/territory list from the page “List of sovereign states” and added a number of additional territories (see Table S1). Using the main page for each country we extracted all pages that link to it, via the “What links here” feature. We then downloaded all pages marked as disputed as those linked to the central page about disputes (“NPOV dispute”) and computed the overlap with the pages above. We ignored pages corresponding to editing and content management (Talk:, User:, User_talk:, Portal:, Portal_talk:, Wikipedia:, Wikipedia_talk:, Category:, Category_talk:, Template:, Template_talk:, File:, File_talk:, Help:, Special:). For German, French and Spanish we used the equivalents of all pages and categories above in the respective langauges.

### Index calculation

We calculated the Wikipedia Dispute Index as:

WDI  =  log (F_dispute_/F_ave_)

Where F_dispute_ is the number of disputed pages linked to a country (D) divided by the total number of pages linking to the country (N), and where F_ave_ is the average of F_dispute_ over all countries considered. Positive values thus denote countries with more disputes than average; negative values the opposite. We also computed another measure whereby each count (N or D) was inversely weighted by the number of countries linked (i.e. to down-weight frequently linked pages), but found little to no difference in the results (see Tables S1, S2).

We ignored those countries/regions where D was smaller than 20. The reasoning was that there were a number pages that appeared for multiple regions that inspection showed had little to do with the particular region considered (see Results & Discussion), meaning that many counts of 20 or fewer were not a true reflection of the region; and regions having values less than this figure show erratic behavior over time (Figure S4) that we believe to be a statistical artifact owing to temporary disputes or those not related to the country. In support of this notion, increasing the D threshold further (see Supporting Information S1; Figure S2) improves the correlation with other indicators.

### Agreement with the other indices

We compared the dispute index to World Bank Policy Research Aggregate Governance Indicators (1996–2008 [13]), including all components (Voice & Accountability, Political Stability No Violence, Government Effectiveness, Regulatory Quality, Rule of Law), and to the 2009 Political Instability Index produced under ViewsWire at the Economist Intelligence Unit [14], also including components (Index score, Underlying Vulnerability, Economic distress). Ideally one would like the indicators to cover exactly the same time period, but the different dates when they are prepared and released makes this impossible. We compared our index from three time points, noticing little difference in the correlation. We chose a time from the middle of our calculations (9 Sep 2008) and roughly matching the apparent date of the two other indices for the plots shown in Figure 2 and Figure S3.

## Supporting Information

Supporting Information S1 A description of how data stringency impacts on how the WDI fits other metrics of geopolitical stability.
(DOC)

Figure S1 Mercator projections of the world colored according the Wikipedia dispute index computed for other languages compared to the English version. Colors traverse the spectrum from red (many more disputes than average) to blue (many fewer than average). Countries having too few disputes to be considered are colored grey. Note that French Territories (e.g. French Guyana, Mayotte, etc.) are colored according to France. Note also that the coloring scheme is relative making differences between maps difficult to interpret particularly owing to the paucity of countries in non-English maps.
(EPS)

Figure S2 Plos showing how correlation with the World Bank indicator improves as one increases the minimum number of disputes allowed (D).
(EPS)

Figure S3 Correlation of the Wikipedia dispute index with two components of the other indicators: the World Bank Policy Research Aggregate Governance Indicator (WGI) on “Voice & Accountability” [13] (left) and the Economist Intelligence Unit (EIU) “Underlying Vulnerability” [14] (right).
(EPS)

Figure S4 Plots showing how values for countries with fewer than 20 disputes (D< = 20) fluctuate drastically over time (left) compared to the G8 countries (right). The countries selected on the left are arbitrary, but all have values of D between 6 and 20.
(EPS)

Table S1 Values of the dispute index for all countries and territories. Values are also given for N D, the ration D/N and weighted equivalents of these values. These values are as for the normal calculation with the difference that counts are weighted by summing the inverse of the number of countries that a page is linked to (instead of 1).
(PDF)

Table S2 Correlation values comparing the Wikipedia dispute index (WDI; red) to components of the World Bank Policy Research Aggregate Governance Indicators[13] (yellow) and b) the Economist Intelligence Unit 2009 political instability index [14] (blue). Five separate tables are shown (sheets in the Excel file) for successively higher values of the minimum number of disputes required for inclusion (D) to demonstrate how correlation improves with stringency. The WDI considered is that for Sep 12 2008, which lies roughly between the dates of the other indicators. The numbers of countries included for each minimum value are: 118 (D< = 20), 71 (50), 42 (100), 26 (150), 17 (200). wWDI denotes the weighted value of the index discussed in the legend to Table S1.
(PDF)
