# Supplementary material for: Content Disputes in Wikipedia Reflect Geopolitical Instability
Source: PLoS One. 2011 Jun 22;6(6):e20902. doi: 10.1371/journal.pone.0020902 (PMC3120813; doi:10.1371/journal.pone.0020902)
Supplement: Supporting Information S1 — A description of how data stringency impacts on how the WDI fits other metrics of geopolitical stability. (DOC) [file pone.0020902.s001.doc]

**Content disputes in Wikipedia reflect geopolitical instability**

*Gordana Apic (1,2), Matthew J. Betts (1) & Robert B. Russell(1)**

1. Cell Networks

University of Heidelberg

Im Neuenheimer Feld 267

69120 Heidelberg

Germany

2. Cambridge Cell Networks Ltd.

St John’s Innovation Centre

Cambridge CB2 0WS

U.K.

* Corresponding author:

Email: robert.russell@bioquant.uni-heidelberg.de

Telephone: +49 6221 54 51362; FAX: +49 6221 54 51487

**Supplementary Information**

***Data stringency and the impact on the results***

In most data analyses one normally considers a threshold of observations required before a trend can be considered genuine. For example, when computing data matrices for biological sequence comparison one typically sets a threshold of 5 observations of a substitution between amino acids to be confident that any ratio measured is likely to reflect reality. Here the main value to consider in this way is the number of pages linking to a country that are disputed (D).

As expected, values of D less than five lead to considerable random variations. However, we noticed that values as high as 20 also give variations in the dispute index that we do not believe to be a reflection of political changes (Figure S4). Inspection (see main text) suggests that several disputed pages are more likely to be the result of irrelevant or background issues not necessarily related to what we attempt to measure here. Values above 20 are more stable (Figure S4)

Restricting the set to countries to those with greater than or equal to 50, 100 or 200 disputes increases the correlation coefficient with the World Bank indicators on political stability from 0.6 to 0.85 (Figure S2), which suggests that the noise level is higher than discussed above, though this is not possible to assess unless one presumes that our index should be a direct match to the instability indices. This is erroneous as a) the other indices are only published annually, which makes dynamics occurring over months impossible to compare, and b) that the indicator here is probably not a precise match to instability/stability metrics (e.g. it matches better to “Voice & Accountability” and “Underlying Vulnerability”; Figure S3).

Similar effects are seen by increasing the threshold for N (the total number of linked pages to a country), for instance to 1000, 5000 or 10 000 pages.
