# Supplementary material for: Content Disputes in Wikipedia Reflect Geopolitical Instability
Source: PLoS One. 2011 Jun 22;6(6):e20902. doi: 10.1371/journal.pone.0020902 (PMC3120813; doi:10.1371/journal.pone.0020902)
Supplement: Table S1 — Values of the dispute index for all countries and territories. Values are also given for N D, the ration D/N and weighted equivalents of these values. These values are as for the normal calculation with the difference that counts are weighted by summing the inverse of the number of countries that a page is linked to (instead of 1). (PDF) [file pone.0020902.s006.pdf]

Code	Name	Total number of linked pages (N)	Number of disputed pages (D)	Fraction disputed (N/D)	Wikipedia dispute index (WDI)	Weighted N	Weighted D	Weighted N/D	Weighted WDI
USA	United States	526233	1782	0.00339	-0.34237	377150.752	997.425	0.00264	0.03451
GBR	United Kingdom	184578	699	0.00379	-0.23054	113993.438	253.517	0.00222	-0.13873
IND	India	91315	582	0.00637	0.29004	60538.848	303.132	0.00501	0.67286
FRA	France	160753	465	0.00289	-0.49995	90542.486	114.143	0.00126	-0.70639
CAN	Canada	137926	464	0.00336	-0.34895	84927.687	191.443	0.00225	-0.12523
DEU	Germany	137200	457	0.00333	-0.35887	69721.142	122.858	0.00176	-0.37149
AUS	Australia	118235	359	0.00304	-0.45147	60161.903	123.865	0.00206	-0.21587
JPN	Japan	101528	314	0.00309	-0.43306	59406.566	96.801	0.00163	-0.44977
ITA	Italy	96061	312	0.00325	-0.3841	46082.011	75.707	0.00164	-0.44157
ISR	Israel	28635	291	0.01016	0.75658	11032.929	82.992	0.00752	1.07984
RUS	Russia	66371	277	0.00417	-0.13336	28251.25	61.008	0.00216	-0.16816
PAK	Pakistan	35300	259	0.00734	0.43083	11627.897	87.355	0.00751	1.07855
ESP	Spain	72349	249	0.00344	-0.32616	29522.882	54.559	0.00185	-0.32391
IRN	Iran	20621	217	0.01052	0.79147	9260.846	54.119	0.00584	0.82736
TUR	Turkey	29242	216	0.00739	0.43756	10772.406	40.886	0.0038	0.39578
IRQ	Iraq	14125	216	0.01529	1.16522	4733.532	50.225	0.01061	1.42382
EGY	Egypt	21085	205	0.00972	0.71233	7501.915	47.231	0.0063	0.90188
BRA	Brazil	56526	205	0.00363	-0.27381	26525.29	42.885	0.00162	-0.4576
AFG	Afghanistan	12699	204	0.01606	1.21448	4751.237	51.443	0.01083	1.44404
NLD	Netherlands	51687	193	0.00373	-0.24463	20634.01	35.732	0.00173	-0.38892
MEX	Mexico	41785	174	0.00416	-0.1356	19536.7	48.01	0.00246	-0.03891
CHN	People's Republic of China	29935	173	0.00578	0.19215	12641.523	46.523	0.00368	0.36494
PHL	Philippines	27874	170	0.0061	0.24599	14072.304	58.971	0.00419	0.49482
CHE	Switzerland	42367	166	0.00392	-0.1965	16501.897	33.504	0.00203	-0.22983
CUB	Cuba	15152	164	0.01082	0.81962	5525.877	38.769	0.00702	1.01015
POL	Poland	97018	159	0.00164	-1.06811	66924.448	40.628	6.07E-04	-1.43713
SWE	Sweden	57103	158	0.00277	-0.54438	23427.387	38.096	0.00163	-0.45181
ZAF	South Africa	36784	149	0.00405	-0.16323	15434.923	40.683	0.00264	0.03116
SYR	Syria	10010	149	0.01489	1.13825	3012.397	27.631	0.00917	1.27817
GRC	Greece	36321	149	0.0041	-0.15057	12019.106	41.138	0.00342	0.29242
SAU	Saudi Arabia	8529	143	0.01677	1.25726	2338.056	27.553	0.01178	1.52876
NZL	New Zealand	45315	139	0.00307	-0.44128	22054.834	38.744	0.00176	-0.37457
BEL	Belgium	37182	139	0.00374	-0.24347	12713.63	25.426	0.002	-0.24493
AUT	Austria	37802	133	0.00352	-0.30413	13787.457	26.88	0.00195	-0.27041
NOR	Norway	42628	130	0.00305	-0.44709	21329.218	19.795	9.28E-04	-1.01269
ARG	Argentina	37521	129	0.00344	-0.3272	14323.844	20.187	0.00141	-0.5949
DNK	Denmark	29957	128	0.00427	-0.10985	11371.17	22.312	0.00196	-0.26399
KOR	South Korea	21875	124	0.00567	0.17282	8981.025	31.391	0.0035	0.31339
SGP	Singapore	17793	121	0.0068	0.35487	6189.9	30.268	0.00489	0.64915
PRT	Portugal	27323	121	0.00443	-0.07405	9331.582	22.234	0.00238	-0.06981
LBN	Lebanon	9046	121	0.01338	1.03135	2797.932	25.109	0.00897	1.25634
MYS	Malaysia	20021	114	0.00569	0.1773	8400.226	30.373	0.00362	0.34727
HKG	Hong Kong	22268	114	0.00512	0.07093	9778.95	37.679	0.00385	0.41085
UKR	Ukraine	22894	104	0.00454	-0.0486	8433.713	26.84	0.00318	0.21962
HUN	Hungary	26594	102	0.00384	-0.21783	10589.622	18.555	0.00175	-0.37715
IDN	Indonesia	20776	100	0.00481	0.00926	7704.554	17.086	0.00222	-0.14155
ROU	Romania	36313	98	0.0027	-0.56932	20607.906	20.84	0.00101	-0.92681
THA	Thailand	19663	95	0.00483	0.01302	7059.18	24.521	0.00347	0.30717
SRB	Serbia	19681	94	0.00478	0.00153	8786.514	19.658	0.00224	-0.13273
IRL	Republic of Ireland	22259	90	0.00404	-0.16505	11513.579	33.015	0.00287	0.11542

FIN	Finland	30981	88	0.00284	-0.51815	11780.785	12.93	0.0011	-0.84492
CHL	Chile	18913	87	0.0046	-0.03606	6267.771	17.037	0.00272	0.06195
VEN	Venezuela	15856	85	0.00536	0.11699	5406.721	19.432	0.00359	0.34123
LKA	Sri Lanka	11478	85	0.00741	0.4401	5758.932	30.333	0.00527	0.72348
COL	Colombia	19087	85	0.00445	-0.06847	7119.358	21.391	0.003	0.16211
VNM	Vietnam	13846	84	0.00607	0.2407	5904.295	18.21	0.00308	0.18824
GEO	Georgia (country)	8140	82	0.01007	0.74781	2798.772	18.447	0.00659	0.94772
BGD	Bangladesh	9292	82	0.00882	0.61545	4453.326	27.892	0.00626	0.89667
BGR	Bulgaria	18754	81	0.00432	-0.09907	7501.204	18.099	0.00241	-0.05724
JOR	Jordan	5706	80	0.01402	1.07839	1523.969	10.245	0.00672	0.96742
MAR	Morocco	11228	78	0.00695	0.37618	3479.366	14.145	0.00407	0.46447
PRK	North Korea	5242	76	0.0145	1.11191	1577.192	14.523	0.00921	1.28204
ETH	Ethiopia	8695	72	0.00828	0.5518	3288.584	14.646	0.00445	0.55569
CZE	Czech Republic	29450	71	0.00241	-0.68213	13054.637	13.449	0.00103	-0.90827
HRV	Croatia	17645	69	0.00391	-0.19846	6032.172	12.935	0.00214	-0.17522
YEM	Yemen	4714	67	0.01421	1.09204	2067.494	11.298	0.00546	0.76022
KWT	Kuwait	4560	66	0.01447	1.11022	1121.047	9.338	0.00833	1.18179
CYP	Cyprus	8982	65	0.00724	0.41705	3131.238	10.98	0.00351	0.31665
BIH	Bosnia and Herzegovina	9087	65	0.00715	0.40543	3562.983	10.961	0.00308	0.18572
kos	Kosovo	3764	64	0.017	1.27129	1054.728	13.361	0.01267	1.60105
TWN	Republic of China	7153	64	0.00895	0.62924	2511.839	12.218	0.00486	0.6439
LBY	Libya	5439	62	0.0114	0.87142	1445.958	6.357	0.0044	0.54277
NGA	Nigeria	13818	61	0.00441	-0.07721	5914.062	13.926	0.00235	-0.08159
DZA	Algeria	9766	61	0.00625	0.26985	3986.024	10.405	0.00261	0.0215
ALB	Albania	8008	61	0.00762	0.46832	2795.484	10.646	0.00381	0.39917
ARM	Armenia	8584	58	0.00676	0.34843	3715.475	11.63	0.00313	0.20306
PER	Peru	18239	55	0.00302	-0.45834	7546.161	10.156	0.00135	-0.64096
NIC	Nicaragua	4980	55	0.01104	0.83979	1514.92	9.049	0.00597	0.84924
KHM	Cambodia	5747	54	0.0094	0.67819	2018.997	10.019	0.00496	0.66385
AZE	Azerbaijan	10982	53	0.00483	0.01192	6977.979	10.254	0.00147	-0.55308
SDN	Sudan	6107	52	0.00851	0.57969	1768.535	10.5	0.00594	0.84325
LTU	Lithuania	10628	52	0.00489	0.02564	3852.756	6.857	0.00178	-0.36154
GHA	Ghana	7162	52	0.00726	0.42034	2505.354	8.769	0.0035	0.31472
NPL	Nepal	10322	51	0.00494	0.03543	6824.944	17.382	0.00255	-0.00319
ARE	United Arab Emirates	6742	51	0.00756	0.46135	2170.139	13.294	0.00613	0.87448
TUN	Tunisia	6547	50	0.00764	0.4709	1825.877	5.647	0.00309	0.1911
SVK	Slovakia	15983	49	0.00307	-0.44182	6418.341	9.88	0.00154	-0.50669
PRI	Puerto Rico	10747	49	0.00456	-0.04492	4449.545	18.015	0.00405	0.4604
MMR	Burma	6003	48	0.008	0.51683	2774.38	7.379	0.00266	0.04015
KEN	Kenya	12296	47	0.00382	-0.22124	5576.834	9.374	0.00168	-0.4187
JAM	Jamaica	8914	47	0.00527	0.10041	3644.951	13.897	0.00381	0.4003
HTI	Haiti	5109	47	0.0092	0.65703	1815.943	10.597	0.00584	0.82595
EST	Estonia	11129	46	0.00413	-0.14303	4953.248	8.157	0.00165	-0.43917
BLR	Belarus	10760	46	0.00428	-0.10931	3259.874	8.354	0.00256	0.00299
QAT	Qatar	4197	45	0.01072	0.81018	926.72	5.96	0.00643	0.9232
ECU	Ecuador	13158	45	0.00342	-0.33248	5579.144	8.118	0.00146	-0.56299
UZB	Uzbekistan	5055	44	0.0087	0.6017	1187.173	6.478	0.00546	0.75887
GTM	Guatemala	6163	44	0.00714	0.40351	2161.933	7.549	0.00349	0.31235
BOL	Bolivia	8619	43	0.00499	0.04512	3216.287	6.164	0.00192	-0.28749
ZWE	Zimbabwe	8294	42	0.00506	0.06002	2571.699	11.914	0.00463	0.5951
MLT	Malta	6762	42	0.00621	0.26424	2278.481	8.342	0.00366	0.35972
ISL	Iceland	9465	42	0.00444	-0.07205	3459.835	5.823	0.00168	-0.41739
SVN	Slovenia	16077	41	0.00255	-0.62593	7283.918	5.097	7.00E-04	-1.29498
PRY	Paraguay	6465	41	0.00634	0.28505	1748.782	8.833	0.00505	0.68154

LVA	Latvia	10651	41	0.00385	-0.2142	3427.173	5.5	0.0016	-0.46504
CRI	Costa Rica	6804	41	0.00603	0.23395	2349.543	11.092	0.00472	0.61395
MNE	Montenegro	4856	40	0.00824	0.54655	1550.058	4.85	0.00313	0.20257
BHR	Bahrain	3194	40	0.01252	0.96549	743.397	7.318	0.00984	1.34884
DOM	Dominican Republic	6525	39	0.00598	0.22581	2588.172	8.69	0.00336	0.27325
pal	Palestinian territories	2188	38	0.01737	1.29248	1000.053	10.364	0.01036	1.40023
MKD	Republic of Macedonia	5635	38	0.00674	0.34647	1877.238	6.215	0.00331	0.25909
URY	Uruguay	8605	37	0.0043	-0.10354	2423.273	5.151	0.00213	-0.18394
SLV	El Salvador	4642	37	0.00797	0.51366	1558.824	8.152	0.00523	0.71634
KAZ	Kazakhstan	7478	36	0.00481	0.00944	2245.096	5.321	0.00237	-0.07516
UGA	Uganda	6401	34	0.00531	0.10779	2099.572	6.129	0.00292	0.13334
HND	Honduras	5200	34	0.00654	0.31559	1754.937	7.722	0.0044	0.54367
PAN	Panama	7432	32	0.00431	-0.10217	2504.501	4.938	0.00197	-0.25921
SOM	Somalia	4273	31	0.00725	0.41956	1157.636	4.431	0.00383	0.40417
LAO	Laos	4184	31	0.00741	0.4406	1258.367	8.958	0.00712	1.02473
TZA	Tanzania	7867	29	0.00369	-0.2575	3266.995	4.622	0.00141	-0.59099
LUX	Luxembourg	7553	28	0.00371	-0.25186	2372.938	3.472	0.00146	-0.55739
TJK	Tajikistan	2907	26	0.00894	0.62886	994.883	2.994	0.00301	0.16364
PNG	Papua New Guinea	5147	26	0.00505	0.05757	2581.631	5.709	0.00221	-0.14438
GUY	Guyana	3509	24	0.00684	0.36061	1138.612	4.095	0.0036	0.34199
COD	Democratic Republic of the Congo	5455	24	0.0044	-0.08059	1687.131	2.515	0.00149	-0.5386
AGO	Angola	5397	24	0.00445	-0.0699	1546.173	3.458	0.00224	-0.13315
RWA	Rwanda	3077	23	0.00747	0.44942	770.133	3.16	0.0041	0.47361
BRB	Barbados	3213	23	0.00716	0.40617	1161.934	7.46	0.00642	0.92148
SEN	Senegal	5306	22	0.00415	-0.13991	1555.413	4.797	0.00308	0.18818
MNG	Mongolia	4434	22	0.00496	0.03962	1847.456	3.463	0.00187	-0.30969
MAC	Macau	3760	22	0.00585	0.20451	925.321	3.171	0.00343	0.29361
KGZ	Kyrgyzstan	3181	22	0.00692	0.37173	1319.256	2.423	0.00184	-0.33029
ERI	Eritrea	2202	22	0.00999	0.73956	675.229	4.45	0.00659	0.94764
cat	Catalonia	4490	21	0.00468	-0.01945	1908.65	5.879	0.00308	0.18694
OMN	Oman	2733	21	0.00768	0.47701	646.425	1.519	0.00235	-0.08378
MDA	Moldova	4977	21	0.00422	-0.12242	2282.079	3.868	0.0017	-0.41031
		Countries with D below threshold							
TTO	Trinidad and Tobago	4182	20	0.00478	0.00283	1479.718	4.897	0.00331	0.25882
MDG	Madagascar	6853	20	0.00292	-0.49107	4043.001	3.321	8.22E-04	-1.13461
abk	Abkhazia	1027	19	0.0185	1.35568	294.403	3.234	0.01099	1.4586
MOZ	Mozambique	4494	19	0.00423	-0.12042	1342.216	2.737	0.00204	-0.22539
MDV	Maldives	1953	19	0.00973	0.71296	798.977	4.966	0.00622	0.88904
MCO	Monaco	3817	19	0.00498	0.04286	901.939	3.297	0.00366	0.35825
FJI	Fiji	4506	19	0.00422	-0.12309	1820.666	2.579	0.00142	-0.59001
ZMB	Zambia	4411	18	0.00408	-0.15585	1250.894	3.246	0.00259	0.0154
TKM	Turkmenistan	2462	18	0.00731	0.42728	661.737	2.254	0.00341	0.28772
CMR	Cameroon	6646	17	0.00256	-0.62292	2271.064	1.762	7.76E-04	-1.19169
BTN	Bhutan	2306	17	0.00737	0.43558	821.107	4.968	0.00605	0.86205
BRN	Brunei	2442	17	0.00696	0.37828	593.354	2.386	0.00402	0.45372
GUM	Guam	4205	16	0.0038	-0.2258	1361.137	3.489	0.00256	0.00318
SLE	Sierra Leone	3760	15	0.00399	-0.17848	1186.473	4.829	0.00407	0.46571
LBR	Liberia	3510	15	0.00427	-0.10968	1118.102	2.391	0.00214	-0.17776
CIV	Ivory Coast	4333	15	0.00346	-0.32033	1910.907	1.796	9.40E-04	-0.9999
BDI	Burundi	2364	15	0.00635	0.28558	618.754	3.777	0.0061	0.87104
LIE	Liechtenstein	1806	14	0.00775	0.48583	477.819	1.253	0.00262	0.02634
IMN	Isle of Man	2983	14	0.00469	-0.01599	1604.072	5.125	0.0032	0.22362
GIB	Gibraltar	3856	14	0.00363	-0.27269	1113.579	2.015	0.00181	-0.34475

VAT	Vatican City	1835	13	0.00708	0.39579	697.004	3.229	0.00463	0.59504
MWI	Malawi	2826	13	0.0046	-0.03603	886.303	0.913	0.00103	-0.9085
BLZ	Belize	2522	13	0.00515	0.07778	808.167	1.865	0.00231	-0.10202
gal	Galicia (Spain)	2278	12	0.00527	0.09949	907.788	3.38	0.00372	0.37651
MUS	Mauritius	3109	12	0.00386	-0.21151	1017.654	3.333	0.00328	0.24841
CYM	Cayman Islands	1237	12	0.0097	0.7101	298.686	5.376	0.018	1.95226
NCL	New Caledonia	2617	11	0.0042	-0.12625	943.585	2.472	0.00262	0.0249
MRT	Mauritania	2037	11	0.0054	0.1243	624.063	0.901	0.00144	-0.57076
BWA	Botswana	3055	11	0.0036	-0.281	1005.086	1.814	0.00181	-0.34739
TLS	East Timor	1975	10	0.00506	0.0599	454.341	0.835	0.00184	-0.32892
TGO	Togo	3146	10	0.00318	-0.40566	1169.606	0.823	7.04E-04	-1.2895
SLB	Solomon Islands	3078	10	0.00325	-0.38381	1135.37	1.445	0.00127	-0.69709
NAM	Namibia	4400	10	0.00227	-0.74114	1646.167	1.025	6.23E-04	-1.41195
CPV	Cape Verde	2419	10	0.00413	-0.14288	881.646	0.971	0.0011	-0.84183
BMU	Bermuda	4035	10	0.00248	-0.65454	1479.463	1.887	0.00128	-0.69487
BHS	The Bahamas	2519	10	0.00397	-0.18339	586.337	2.854	0.00487	0.64454
GIN	Guinea	3312	9	0.00272	-0.56244	1046.196	2.015	0.00193	-0.28281
GAB	Gabon	2875	9	0.00313	-0.42094	790.446	0.916	0.00116	-0.79074
FLK	Falkland Islands	1520	9	0.00592	0.2164	523.232	1.644	0.00314	0.20709
nkr	Nagorno-Karabakh Republic	753	8	0.01062	0.80102	151.78	1.949	0.01284	1.61474
NER	Niger	2442	8	0.00328	-0.37549	871.884	0.504	5.78E-04	-1.48557
MNP	Northern Mariana Islands	1165	8	0.00687	0.3646	238.323	1.913	0.00803	1.14475
LSO	Lesotho	1509	8	0.0053	0.10588	559.367	1.394	0.00249	-0.02454
tra	Transnistria	686	7	0.0102	0.76067	244.324	1.335	0.00546	0.76013
SUR	Suriname	2452	7	0.00285	-0.51311	735.126	1.347	0.00183	-0.33259
JEY	Jersey	1766	7	0.00396	-0.18492	681.39	2.924	0.00429	0.51842
GRD	Grenada	1676	7	0.00418	-0.13262	511.045	1.49	0.00292	0.13198
GNQ	Equatorial Guinea	1819	7	0.00385	-0.21449	440.373	0.388	8.82E-04	-1.06367
GGY	Guernsey	1284	7	0.00545	0.13381	440.353	1.704	0.00387	0.41508
FRO	Faroe Islands	2085	7	0.00336	-0.35097	759.327	1.564	0.00206	-0.21515
CAF	Central African Republic	2237	7	0.00313	-0.42134	627.895	1.102	0.00176	-0.37539
AND	Andorra	1457	7	0.0048	0.00742	430.878	1.041	0.00242	-0.05588
bas	Basque Country (autonomous	1527	6	0.00393	-0.19366	592.431	0.942	0.00159	-0.47461
WSM	Samoa	2452	6	0.00245	-0.66726	911.571	1.829	0.00201	-0.24167
VUT	Vanuatu	1358	6	0.00442	-0.07637	487.572	0.935	0.00192	-0.28643
VCT	Saint Vincent and the Grenadines	1005	6	0.00597	0.22466	282.884	2.487	0.00879	1.2356
MSR	Montserrat	931	6	0.00644	0.30114	221.155	1.243	0.00562	0.78814
COG	Republic of the Congo	2363	6	0.00254	-0.63029	631.331	0.64	0.00101	-0.92362
BFA	Burkina Faso	4830	6	0.00124	-1.3452	3031.884	1.361	4.49E-04	-1.73914
ATG	Antigua and Barbuda	1105	6	0.00543	0.1298	310.053	2.194	0.00708	1.01869
ANT	Netherlands Antilles	1420	6	0.00423	-0.12101	311.118	0.384	0.00123	-0.72717
ncy	Northern Cyprus	686	5	0.00729	0.4242	129.509	0.825	0.00637	0.91361
VGB	British Virgin Islands	1097	5	0.00456	-0.04526	275.802	1.904	0.0069	0.99381
TON	Tonga	1916	5	0.00261	-0.60292	649.537	0.513	7.90E-04	-1.17382
TCD	Chad	2477	5	0.00202	-0.85973	759.84	0.868	0.00114	-0.80535
TCA	Turks and Caicos Islands	985	5	0.00508	0.06244	173.869	0.342	0.00197	-0.26248
SYC	Seychelles	1604	5	0.00312	-0.42518	540.117	0.682	0.00126	-0.70515
STP	Sao Tome and Principe	940	5	0.00532	0.1092	353.408	0.325	9.20E-04	-1.02089
MLI	Mali	4407	5	0.00113	-1.43587	1791.425	0.464	2.59E-04	-2.28809
GRL	Greenland	3318	5	0.00151	-1.15204	1362.243	0.446	3.27E-04	-2.05529
GMB	The Gambia	1307	5	0.00383	-0.22041	453.118	0.492	0.00109	-0.85592

DMA	Dominica	1318	5	0.00379	-0.22879	451.094	1.767	0.00392	0.42715
DJI	Djibouti	1386	5	0.00361	-0.2791	291.888	0.538	0.00184	-0.32734
BEN	Benin	2597	5	0.00193	-0.90703	870.662	0.53	6.08E-04	-1.43484
TUV	Tuvalu	905	4	0.00442	-0.076	221.921	0.282	0.00127	-0.69885
SMR	San Marino	1614	4	0.00248	-0.65454	394.652	0.351	8.90E-04	-1.05419
PYF	French Polynesia	1632	4	0.00245	-0.66563	726.343	0.295	4.07E-04	-1.83778
PLW	Palau	1680	4	0.00238	-0.69462	435.568	0.232	5.32E-04	-1.5693
PCN	Pitcairn Islands	854	4	0.00468	-0.018	128.294	0.225	0.00175	-0.37596
NRU	Nauru	832	4	0.00481	0.0081	225.973	0.441	0.00195	-0.27021
LCA	Saint Lucia	1108	4	0.00361	-0.27838	388.72	0.9	0.00232	-0.09849
GNB	Guinea-Bissau	1487	4	0.00269	-0.57258	335.718	1.161	0.00346	0.30292
FSM	Federated States of Micronesia	726	4	0.00551	0.14438	199.808	0.336	0.00168	-0.41967
AIA	Anguilla	806	4	0.00496	0.03985	197.786	1.12	0.00566	0.79614
ABW	Aruba	1253	4	0.00319	-0.40136	332.024	0.351	0.00106	-0.88297
aad	Akrotiri and Dhekelia	277	3	0.01083	0.82023	25.093	0.269	0.01073	1.43473
WLF	Wallis and Futuna	630	3	0.00476	-0.00147	98.72	0.157	0.00159	-0.47224
VIR	United States Virgin Islands	1108	3	0.00271	-0.56606	348.467	0.427	0.00123	-0.73496
SWZ	Swaziland	1604	3	0.00187	-0.936	445.97	0.694	0.00156	-0.49516
SPM	Saint Pierre and Miquelon	672	3	0.00446	-0.06601	105.86	0.169	0.0016	-0.46911
SGS	South Georgia and the South Sandwich Islands	732	3	0.0041	-0.15153	328.853	0.101	3.06E-04	-2.12242
NFK	Norfolk Island	1054	3	0.00285	-0.5161	280.223	0.355	0.00127	-0.70216
KNA	Saint Kitts and Nevis	843	3	0.00356	-0.29271	230.848	1.3	0.00563	0.79034
COM	Comoros	981	3	0.00306	-0.44432	313.207	0.185	5.90E-04	-1.46603
COK	Cook Islands	1268	3	0.00237	-0.70094	393.978	0.638	0.00162	-0.45583
chr	Christmas Island	758	2	0.00264	-0.5919	245.607	0.216	8.79E-04	-1.0669
MHL	Marshall Islands	1873	2	0.00107	-1.49651	535.374	0.077	1.44E-04	-2.87818
KIR	Kiribati	815	2	0.00245	-0.6644	262.591	0.085	3.25E-04	-2.06064
IOT	British Indian Ocean Territory	390	2	0.00513	0.07264	52.63	0.07	0.00134	-0.6485
coc	Cocos (Keeling) Islands	580	1	0.00172	-1.01739	88.426	0.083	9.42E-04	-0.99734
NIU	Niue	681	1	0.00147	-1.17792	140.267	0.071	5.09E-04	-1.61288
TKL	Tokelau	426	0	0	0	105.137	0	0	0
HMD	Heard Island and McDonald Islands	153	0	0	0	59.965	0	0	0
