# Supplementary material for: Content Disputes in Wikipedia Reflect Geopolitical Instability
Source: PLoS One. 2011 Jun 22;6(6):e20902. doi: 10.1371/journal.pone.0020902 (PMC3120813; doi:10.1371/journal.pone.0020902)
Supplement: Table S2 — Correlation values comparing the Wikipedia dispute index (WDI; red) to components of the World Bank Policy Research Aggregate Governance Indicators[13] (yellow) and b) the Economist Intelligence Unit 2009 political instability index [14] (blue). Five separate tables are shown (sheets in the Excel file) for successively higher values of the minimum number of disputes required for inclusion (D) to demonstrate how correlation improves with stringency. The WDI considered is that for Sep 12 2008, which lies roughly between the dates of the other indicators. The numbers of countries included for each minimum value are: 118 (D< = 20), 71 (50), 42 (100), 26 (150), 17 (200). wWDI denotes the weighted value of the index discussed in the legend to Table S1. (PDF) [file pone.0020902.s007.pdf]

D>=20	WDI	wWDI	Underlying vulnerability	Economic distress	Score (2009)	Score (2007)	Control of Corruption	Government Effectiveness	Political Stability	Regulatory Quality	Rule of Law	Voice and Accountability
WDI	--	0.933	0.310	0.054	0.248	0.233	-0.389	-0.440	-0.429	-0.444	-0.386	-0.613
wWDI	--	--	0.351	0.117	0.307	0.295	-0.406	-0.437	-0.486	-0.443	-0.398	-0.598
Underlying vulnerability	--	--	--	0.382	0.892	0.844	-0.772	-0.747	-0.771	-0.648	-0.794	-0.551
Economic distress	--	--	--	--	0.758	0.445	-0.327	-0.340	-0.316	-0.282	-0.317	-0.149
Score (2009)	--	--	--	--	--	0.816	-0.705	-0.694	-0.699	-0.597	-0.716	-0.464
Score (2007)	--	--	--	--	--	--	-0.716	-0.680	-0.722	-0.583	-0.721	-0.500
Control of Corruption	--	--	--	--	--	--	--	0.954	0.754	0.884	0.961	0.774
Government Effectiveness	--	--	--	--	--	--	--	--	0.742	0.939	0.961	0.796
Political Stability	--	--	--	--	--	--	--	--	--	0.699	0.784	0.623
Regulatory Quality	--	--	--	--	--	--	--	--	--	--	0.912	0.854
Rule of Law	--	--	--	--	--	--	--	--	--	--	--	0.776
Voice and Accountability	--	--	--	--	--	--	--	--	--	--	--	--

D>=50	WDI	wWDI	Underlying vulnerability	Economic distress	Score (2009)	Score (2007)	Control of Corruption	Government Effectiveness	Political Stability	Regulatory Quality	Rule of Law	Voice and Accountability
WDI	--	0.959	0.477	0.130	0.406	0.420	-0.554	-0.631	-0.549	-0.626	-0.580	-0.747
wWDI	--	--	0.523	0.173	0.459	0.469	-0.552	-0.611	-0.602	-0.604	-0.575	-0.742
Underlying vulnerability	--	--	--	0.373	0.891	0.871	-0.783	-0.740	-0.787	-0.679	-0.799	-0.601
Economic distress	--	--	--	--	0.753	0.402	-0.322	-0.324	-0.304	-0.244	-0.295	-0.172
Score (2009)	--	--	--	--	--	0.817	-0.714	-0.685	-0.709	-0.604	-0.712	-0.514
Score (2007)	--	--	--	--	--	--	-0.744	-0.690	-0.766	-0.602	-0.738	-0.535
Control of Corruption	--	--	--	--	--	--	--	0.964	0.783	0.923	0.967	0.803
Government Effectiveness	--	--	--	--	--	--	--	--	0.765	0.949	0.956	0.822
Political Stability	--	--	--	--	--	--	--	--	--	0.720	0.813	0.638
Regulatory Quality	--	--	--	--	--	--	--	--	--	--	0.929	0.875
Rule of Law	--	--	--	--	--	--	--	--	--	--	--	0.806
Voice and Accountability	--	--	--	--	--	--	--	--	--	--	--	--

D>=100	WDI	wWDI	Underlying vulnerability	Economic distress	Score (2009)	Score (2007)	Control of Corruption	Government Effectiveness	Political Stability	Regulatory Quality	Rule of Law	Voice and Accountability
WDI	--	0.977	0.614	0.174	0.516	0.502	-0.677	-0.746	-0.759	-0.775	-0.717	-0.843
wWDI	--	--	0.641	0.182	0.538	0.543	-0.689	-0.736	-0.781	-0.766	-0.722	-0.832
Underlying vulnerability	--	--	--	0.366	0.880	0.870	-0.811	-0.784	-0.859	-0.702	-0.827	-0.654
Economic distress	--	--	--	--	0.764	0.421	-0.238	-0.204	-0.263	-0.092	-0.194	-0.150
Score (2009)	--	--	--	--	--	0.821	-0.685	-0.650	-0.732	-0.538	-0.674	-0.533
Score (2007)	--	--	--	--	--	--	-0.806	-0.776	-0.819	-0.658	-0.794	-0.603
Control of Corruption	--	--	--	--	--	--	--	0.968	0.854	0.914	0.965	0.855
Government Effectiveness	--	--	--	--	--	--	--	--	0.858	0.943	0.971	0.880
Political Stability	--	--	--	--	--	--	--	--	--	0.788	0.877	0.747
Regulatory Quality	--	--	--	--	--	--	--	--	--	--	0.938	0.925
Rule of Law	--	--	--	--	--	--	--	--	--	--	--	0.867
Voice and Accountability	--	--	--	--	--	--	--	--	--	--	--	--

D>=150	WDI	wWDI	Underlying vulnerability	Economic distress	Score (2009)	Score (2007)	Control of Corruption	Government Effectiveness	Political Stability	Regulatory Quality	Rule of Law	Voice and Accountability
WDI	--	0.981	0.630	0.163	0.525	0.505	-0.682	-0.737	-0.734	-0.783	-0.712	-0.874
wWDI	--	--	0.623	0.131	0.502	0.506	-0.656	-0.701	-0.740	-0.746	-0.683	-0.833
Underlying vulnerability	--	--	--	0.336	0.871	0.883	-0.760	-0.742	-0.893	-0.665	-0.789	-0.643
Economic distress	--	--	--	--	0.756	0.385	-0.115	-0.085	-0.208	-0.021	-0.091	-0.135
Score (2009)	--	--	--	--	--	0.816	-0.590	-0.563	-0.731	-0.477	-0.598	-0.520
Score (2007)	--	--	--	--	--	--	-0.758	-0.731	-0.809	-0.579	-0.737	-0.538
Control of Corruption	--	--	--	--	--	--	--	0.980	0.832	0.914	0.965	0.862
Government Effectiveness	--	--	--	--	--	--	--	--	0.821	0.949	0.973	0.900
Political Stability	--	--	--	--	--	--	--	--	--	0.737	0.847	0.698
Regulatory Quality	--	--	--	--	--	--	--	--	--	--	0.932	0.925
Rule of Law	--	--	--	--	--	--	--	--	--	--	--	0.861
Voice and Accountability	--	--	--	--	--	--	--	--	--	--	--	--

D>=200	WDI	wWDI	Underlying vulnerability	Economic distress	Score (2009)	Score (2007)	Control of Corruption	Government Effectiveness	Political Stability	Regulatory Quality	Rule of Law	Voice and Accountability
WDI	--	0.981	0.762	0.038	0.487	0.552	-0.741	-0.792	-0.813	-0.833	-0.833	-0.860
wWDI	--	--	0.751	-0.003	0.455	0.555	-0.686	-0.733	-0.798	-0.773	-0.772	-0.795
Underlying vulnerability	--	--	--	0.409	0.845	0.827	-0.751	-0.709	-0.917	-0.666	-0.746	-0.667
Economic distress	--	--	--	--	0.833	0.466	-0.048	0.000	-0.182	0.061	-0.044	-0.006
Score (2009)	--	--	--	--	--	0.775	-0.484	-0.432	-0.663	-0.371	-0.479	-0.411
Score (2007)	--	--	--	--	--	--	-0.656	-0.614	-0.767	-0.469	-0.610	-0.424
Control of Corruption	--	--	--	--	--	--	--	0.975	0.822	0.907	0.974	0.865
Government Effectiveness	--	--	--	--	--	--	--	--	0.789	0.955	0.983	0.917
Political Stability	--	--	--	--	--	--	--	--	--	0.696	0.811	0.691
Regulatory Quality	--	--	--	--	--	--	--	--	--	--	0.952	0.967
Rule of Law	--	--	--	--	--	--	--	--	--	--	--	0.936
Voice and Accountability	--	--	--	--	--	--	--	--	--	--	--	--
